# Sustainable heteroatom doped biochar for methylene blue adsorption with structure function insights

**DOI:** 10.1038/s41598-026-48042-z

**Published:** 2026-04-22

**Authors:** Vivian Fayez Lotfy, Altaf Halim Basta

**Affiliations:** Cellulose and Paper Department, National Research Centre, Dokki, Giza, 12622 Egypt

**Keywords:** Agro-based biochar, Nitrogen doping, Phosphoric acid activation, Methylene blue adsorption, Adsorption isotherms, Adsorption kinetics, Chemistry, Environmental sciences

## Abstract

This study investigates the enhancement of agro-derived biochars as sustainable, low-cost adsorbents for dye-contaminated wastewater treatment. Four agricultural residues—rice straw (RS), date palm fiber (DP), sugarcane bagasse (SCB), and giant reed (*Arundo donax* L., GR)—were evaluated as biochar precursors. RS, the most carbon-rich, exhibited limited surface reactivity, prompting further modification via hydrothermal nitrogen (N) doping and phosphoric acid (H₃PO₄) activation. The modified biochars were characterized for surface chemistry, porosity, and morphology using elemental analysis, FTIR, SEM, and iodine number, and their methylene blue adsorption performance was systematically investigated under varying pH, adsorbent dosage, and initial dye concentration. The novelty of this work lies in the systematic comparison of hydrothermal N-doping, microwave-assisted treatment, and low-cost phosphoric acid activation on the same precursor, elucidating how complementary acidic and basic surface functionalization strategies govern adsorption performance. Adsorption was governed by electrostatic interactions, π–π stacking, hydrogen bonding, and surface complexation, influenced by the biochar’s porosity, surface functional groups, and chemical composition. Hydrothermally N-doped RS (N-RS-HT) exhibited enriched nitrogen content (3.56%), increased basic site density (1.495 mmol g⁻^1^), specific surface area (434.9 × 10^3^ m^2^ kg⁻^1^), and adsorption capacity (136.9 ± 6.9 mg g⁻^1^), while RS-P showed higher acidity (8.2 mmol g⁻^1^), surface area (462.8 × 10^3^ m^2^ kg⁻^1^), and MB uptake (145.8 ± 7.3 mg g⁻^1^). Adsorption followed Langmuir isotherms, pseudo-second-order kinetics, and was spontaneous and endothermic, demonstrating complementary strategies for designing high-performance biochar for practical wastewater treatment applications.

## Introduction

Biochar, a carbon-rich porous material produced from the pyrolysis of biomass under oxygen-limited conditions, has attracted growing interest for its multifunctional applications in environmental management, agriculture, and clean technologies^1,2^. Owing to its surface area, tunable porosity, and diverse surface functional groups, biochar has been utilized in soil improvement, carbon sequestration, waste valorization, and emerging industrial uses such as catalyst supports, energy storage, rubber filler and nanocomposites^3,4^. Recently, its potential as a low-cost adsorbent for water treatment targeting heavy metals, dyes, pesticides, and organic contaminants has received significant attention^5,6^. New strategies for managing agriculture waste involve using it as a renewable resource for manufacturing advanced nanomaterials^7,8^, delivery systems^9^, agricultural purpose^10^, liquid crystal nanocomposites^11,12^, functional lignocellulosic materials^10,13–15,70^, construction materials^16^, and carbon nanostructures^17^.

Adsorption is a versatile, cost‑effective method for removing a variety of pollutants. Cationic dyes like methylene blue are efficiently captured via electrostatic attraction, π–π interactions, and tailored surface functionalities, whereas other contaminants often require different surface chemistries or pore structures^18^. The adsorption performance of pristine biochar, however, is often constrained by limited active sites, low polarity, and weak affinity for certain pollutants. To address these drawbacks, various modification strategies have been developed. Physical treatments (e.g., activation, ball milling) enhance porosity, while chemical treatments (e.g., acid/alkali modification) introduce functional groups that improve electrostatic interactions^19,5^. Metal or metal oxide impregnation (e.g., Fe, Mg, Mn, Al) enhances affinity for heavy metals and oxyanions, whereas magnetic biochar facilitates both adsorption and easy separation from water (Qambrani et al., 2017;^20^. Metal doping improves adsorption but may cause contamination, high cost, and leaching, while heteroatom functionalization offers a safer, low-cost alternative with effective performance. Advanced composites with clays, polymers, or nanomaterials further combine adsorption with photocatalysis or oxidation processes^3,21,22^.

Among these strategies, heteroatom doping the incorporation of elements such as nitrogen (N), sulfur (S), phosphorus (P), and boron (B) has shown exceptional promise. Heteroatoms alter the electronic structure and surface chemistry of biochar, enhancing adsorption through the introduction of new functional groups and changes in surface charge^23^. For example, N-doping introduces groups that strengthen interactions with metals and acidic organics^24^, S-doping provides thiol and sulfonic groups with strong metal affinity^25^, and P-doping increases cation exchange capacity and oxyanion binding^26^. Doping often improves porosity and creates more active sites^27^, while multi-heteroatom or metal–heteroatom composites offer synergistic effects for broad-spectrum contaminant removal^20^.

Methylene blue is the popular name for the aromatic heterocyclic, organic chloride salt of 3,7-bis(dimethylamino)phenothiazin-5-ium that has a deep blue color^28^. Due to its cardioprotective, antimalarial, depressive, and antioxidant qualities, it is employed in dyeing and medication. MB has mostly been utilized for a number of therapeutic and diagnostic operations in human and veterinary medicine^29^. Heinrich Caro synthesized it for the first time in 1800^30^. Above a certain concentration (25 mg kg^−1^), MB is toxic, carcinogenic, and non-biodegradable, and it can have detrimental impacts on the environment and pose a major risk to human health^28^. Methylene blue was selected as a widely accepted model cationic dye due to its well-known adsorption behavior, stable structure, strong chromophore signal, and relevance to textile wastewater, enabling reliable comparison with literature and mechanistic evaluation.

Removing cationic dyes typically requires adsorbents with tailored surface charge and specific functional groups to enhance electrostatic attraction and π–π interactions, whereas adsorption of gases or small aqueous pollutants is often dominated by surface area and pore structure. Literature reports a broad range of adsorbent materials such as activated carbon, zeolites, silica gel, clay, biomass‑derived carbons, metal–organic frameworks (MOFs), graphene‑based nanomaterials and functionalized polymers like chitosan whose surface chemistry and porosity govern removal efficiency across different pollutants^31^. Biomass‑based adsorbents have received recent attention for gaseous contaminant capture due to their low cost, high porosity, and tunable surface functionality^32,33^.

This study presents a comprehensive and unprecedented evaluation of heteroatom-doped biochar prepared from the optimum samples of four agricultural wastes: rice straw (RS), date palm fiber (DP), sugarcane bagasse (SCB), and giant reed (GR, *Arundo donax* L.). Two sustainable functionalization routes (hydrothermal and microwave) are directly compared, providing new insight into how processing pathways influence doping efficiency and surface chemistry. The optimized N-doped biochar is further benchmarked against conventional phosphoric-acid activation, clearly demonstrating the enhanced performance imparted by heteroatom incorporation. By integrating detailed physicochemical characterization (surface functionality, elemental composition, FTIR, and SEM) with adsorption studies under varying conditions (pH, initial dye concentration, adsorbent dosage, and temperature) and kinetic modeling, the work establishes new structure–function relationships governing methylene blue removal. These elements underscore a distinct and multifaceted novelty not previously addressed in the literature.

## Materials and methods

### Materials

Biochar was produced from four Egyptian agricultural by-products: rice straw (RS), date palm fiber (DP), sugarcane bagasse (SCB), and giant reed (GR, *Arundo donax *L.). These precursors were collected from fields. These precursors were collected from agricultural fields in the Nile Delta region of Egypt. For chemical modification, phosphoric acid (H_3_PO_4_; 85%, El-Nasr Pharmaceutical Chemistry Co., ADWIC) was used as an activating agent, while pure hexamethylenetetramine (TMAOH, VEB Laborchemie Apolda, Germany) was employed as a nitrogen source. Sulfuric acid (H₂SO₄, 98%) and sodium hydroxide (NaOH), were supplied by Merck (Germany). Methylene blue (MB, C₁₆H₁₈N₃SCl) was employed as the model dye in the adsorption experiments. The stock solution was prepared at a concentration of 1000 mg L⁻^1^. MB has a maximum absorption wavelength of 664 nm, molecular weight 319.85 g mol⁻^1^, and is listed under Color Index (CI) number 52015. Iodine, used as an additional probe molecule, was obtained from El-Nasr Pharmaceutical Chemical.

### Chemical constituents analyses

The chemical compositions of agro-fiber precursors are now consistently presented as follows: rice straw (RS), date palm fiber (DP), sugarcane bagasse (SCB), and giant reed (GR, *Arundo donax *L.). Holocellulose was prepared by delignification with sodium chlorite in an acetate buffer (pH ~ 4) at 75–80 °C for 4–5 h, until the fibers turned white, followed by washing, drying, and weighing^34^. From this fraction, α-cellulose was obtained through extraction with 17.5% NaOH at 20 °C for 30 min, then thoroughly washed, neutralized, and dried (The Institute of Paper Chemistry (1952), Institute Method No. 424). Hemicellulose was separated from holocellulose by sequential treatment with 8 wt.% NaOH in two steps, forming a homogeneous paste, allowed to swell at 20 °C for 35 min, then diluted with distilled water, suction-filtered, and repeatedly washed to neutrality. Lignin content was determined by two-step acid hydrolysis using 72% H₂SO₄ at 20 °C followed by 3% H₂SO₄ at 120 °C, with the acid-insoluble residue representing lignin (Method No. 428, 1951;^35^). Ash was quantified by sequential combustion at 450 °C for 1 h and 800 °C for 45 min, expressed as a percentage of dry weight. All measurements were conducted in triplicate, and results were reported as percentages of oven-dried biomass**.**

### Preparation of biochar

The foregoing four agricultural wastes: were used as precursors for biochar production. The raw materials were collected from Egyptian fields, washed with distilled water, and oven-dried at 105 °C. The dried samples were ground using knife mills equipped with 2 mm sieves and used without further fractionation. Pyrolysis was conducted in a muffle furnace at 400 °C for 1 h under oxygen-limited conditions. The average biochar yields obtained from RS, DP, SCB, and GR were 48.2, 60.8, 57.3, and 46.7%, respectively. Biochar yield (%) was calculated using the following equation:

$$Biochar yield \left( \% \right) = \frac{{\text{Weight of dried biochar}}}{{\text{Weight of dry precursor}}} \times 100$$

### Surface modification and chemical activation

Based on adsorption study, the surface modification of rice straw (RS) and sugarcane bagasse (SCB) biochars was performed following previously reported methods with slight modifications. A TMAOH solution was added to the dried biochar in a 1:1 ratio, stirred thoroughly, and left to stand for 24 h to facilitate the introduction of surface functional groups. After this step, two different approaches were applied for nitrogen functionalization. In the first approach (hydrothermal method)^36,37^, the mixture was transferred to a 200 mL Teflon-lined autoclave and heated in a static electric oven at 180 °C for 3 h. The solid product was recovered by filtration, repeatedly washed with distilled water, and oven-dried. The obtained N-doped biochars were denoted as N-RS-H and N-SCB-H. In the second approach (microwave-assisted method)^38^, the mixture was transferred to a 150 mL beaker and subjected to microwave irradiation in a domestic microwave oven (Samsung MS40J5133BG/40L, 1000 W) for 15 min. During microwave oven treatment, the residual water molecules were evaporated, leading to the formation of a gray precipitate. The solid product was similarly repeatedly washed and dried. The resulting N-doped biochars were labeled as N-RS-M and N-SCB-M.

Based on the MB adsorption study, chemical modification using phosphoric acid was applied exclusively to rice straw (RS) biochar. Phosphoric acid was employed as the activating agent and compared with the nitrogen-doping technique at a ratio of 3:1 (activator: dried biochar). The activated precursor was then pyrolyzed in a muffle furnace at 450 °C for 60 min under an oxygen-free atmosphere. The activation conditions were selected based on preliminary tests and previous literature to optimize porosity while minimizing burn-off^39^. The resulting activated biochar was designated as RS-P.

### Characterization of biochar and surface-modified biochar

#### Methylene blue adsorption performance

Stock solutions of methylene blue (MB, 1000 mg L⁻^1^) were diluted to obtain working solutions with concentrations ranging from 50 to 300 mg L⁻^1^. Batch adsorption experiments were carried out by mixing 25 mg of biochar with 10 mL of dye solution in 100 mL reagent bottles. The suspensions were agitated at 100 rpm for 24 h at 30 °C to reach equilibrium. After equilibration, the mixtures were centrifuged, and the residual MB concentration in the supernatant was determined spectrophotometrically at 664 nm using a UV–Vis single-beam spectrophotometer (UV1720, USA). The equilibrium adsorption capacity, Qe (mg g⁻^1^), was calculated using:

$$Q_{e} (mg/g) = \frac{{\left( {C_{o} - C_{e} } \right)V}}{W}$$

where C_o_ and C_e_ (mg L⁻1) are the liquid-phase concentrations of the dye at initial and equilibrium, V (L): volume of the dye solution, W (g): weight of biochar.

Adsorption isotherms were analyzed using the Langmuir, Freundlich, Temkin, and Dubinin–Radushkevich (D–R) models^40–43^. The models and their equations are summarized in the following table. For Langmuir, a plot of Ce/q_e_ versus Ce gives a straight line, from which Qm and b are calculated. For Freundlich, plotting log q_e_ versus log Ce yields K_F_ and n. The Temkin constants A_T_ and B are obtained from a plot of qe versus ln Ce while the D–R constants q_m_ and β are determined from ln q_e_ versus ε^2^. The applicability of each isotherm model was evaluated by comparing the correlation coefficients (R^2^) obtained from the linear plots.

**Table:** 

Isotherm model	Equation form	Refs.
Langmuir isotherm	$\frac{{C_{e} }}{{q_{e} }} = \frac{1}{{{\mathrm{b}}q_{m} }} + \frac{{C_{e} }}{{q_{m} }}$ $R_{L} = { }\frac{1}{{1 + {\mathrm{bC}}_{0} }}$	^42^
Freundlich isotherm	$\log qe = \log K_{F} + \frac{1}{n} log C_{e}$	^41^
Temkin isotherm	$q_{e} = \frac{RT}{B}{\text{Ln A}}_{T} { } + \frac{RT}{B}{\text{Ln C}}_{e}$	^43^
D-R isotherm	$Ln q_{e} = Ln q_{m} - \beta \varepsilon^{2}$ $\varepsilon = RT Ln \left( {1 + \frac{1}{{C_{e} }}} \right)$ $E_{DR} = \frac{1}{{\sqrt { - 2\beta } }}$	Dubinin & Radushkevich

Where, q_e_ is the amount of dye adsorbed at equilibrium, C_e_ is the equilibrium concentration of the adsorbate (dye), q_m_ (mg g^−1^) is the maximum adsorption capacity, b is Langmuir isotherm constant (L/mg) and R_L_ is a separation factor (dimensionless Index). The constant K_f_ is an approximate indicator of adsorption capacity, while n is adsorption intensity. A_T_ is Temkin isotherm constant (L/g), B is the Temkin constant (J/mol), R is universal gas constant (8.314 J/mol K) and T is absolute temperature. β is the activity coefficient useful in obtaining the mean sorption energy E (kJ/mol) and Ɛ is the Polanyi potential.

#### Specific surface area

For all adsorption isotherms of methylene blue on modified biochar, the linear plot of Ce/q_e_ versus Ce was obtained. The slope of this line corresponds to 1/q_m_, while the intercept equals 1/Kq_m_. The high correlation coefficient confirms that the Langmuir isotherm provides an appropriate description of the adsorption behavior of methylene blue onto the adsorbent. The specific surface area (S_MB_) of the adsorbent was then calculated using the following equation^44^:

$$S_{MB} = \frac{{Q_{e} \times a_{MB} \times N_{A} \times 10^{ - 20} }}{M}$$

**S**_MB_: the specific surface area, expressed in 10^−3^ km^2^ kg^−1^, Qe: the amount of methylene blue adsorbed at monolayer coverage, in mg g⁻^1^.a_MB_ is the surface area occupied by a single methylene blue molecule, reported as 197.2 Å^2^. N_A_ is Avogadro’s number, 6.02 × 10^23^ mol⁻^1^. M is the molecular weight of methylene blue, equal to 373.9 g mol⁻^1^.

#### Iodine value

The iodine number test, considered a fundamental method for assessing microporosity in carbon-based materials, was determined at room temperature (~ 25 °C) following the ASTM D4607-94 standard procedure^45^. This method is based on the adsorption of iodine from aqueous solution, where the amount adsorbed at equilibrium is directly related to the accessible surface area and micropore volume of the adsorbent, thereby serving as an indicator of the degree of activation and pore development.

### Surface chemistry (acidic and basic groups)

The surface acidity and basicity of the modified biochars were evaluated following the method described in^46^. In each test, 150 mg of nitrogen-doped or chemically activated biochar was mixed with 15 mL of either 0.1 M HCl or 0.1 M NaOH in a closed flask and stirred for 24 h at room temperature. The suspensions were subsequently titrated with 0.1 M NaOH or 0.1 M HCl, respectively, and the residual concentration of the acid or base was used to quantify the surface acidic and basic sites of the bio-adsorbents.

### Adsorption batch experiment

Batch adsorption experiments were performed in Erlenmeyer flasks using MB solutions with initial concentrations of 50–300 mg/L. Experimental conditions were varied by adjusting pH (4–10), adsorbent dosage (25–100 mg), dye volume (10–50 mL), and temperature (25–35 °C). For each run, 10 mL of MB solution at the desired concentration was placed in a flask, and the pH was controlled using 0.1 M HCl or NaOH. The required amount of adsorbent was then added, and the mixtures were agitated at 100 RPM. After equilibrium was reached, the suspensions were centrifuged to separate the solid and liquid phases. The equilibrium adsorption capacity (Qe, mg/g) was calculated using the last standard equation, while the percentage removal was determined according to the following expression:

$${\mathrm{Re}} moval efficiency \left( \% \right) = \frac{{\left( {C_{o} - C_{e} } \right)}}{{C_{o} }} \times 100$$

where C_o_ and C_e_ (mg/L) are the liquid-phase concentrations of the dye at initial and equilibrium.

Thermodynamic parameters were evaluated using the distribution coefficient (K_D_) derived from equilibrium concentrations of MB in solution and on the biochar surface^39^. The Gibbs free energy change (ΔG°) was calculated using the following equation, while the enthalpy (ΔH°) and entropy (ΔS°) were obtained from the slope and intercept of the Van’t Hoff plot of ln K_D_ versus 1/T. This approach enables assessment of the spontaneity and heat effects governing MB adsorption.

$$K_{D} = \frac{{Q_{e} }}{{C_{e} }}$$

$$\Delta G^{o} = - RT lnK_{D}$$

$$lnK_{D} = \left( {\frac{{\Delta S^{o} }}{R}} \right) - \left( {\frac{{\Delta H^{o} }}{RT}} \right)$$

where R is the universal gas constant (8.314 J mol⁻1 K⁻1) and T is the absolute temperature (K), K_D_ is the distribution coefficient of the adsorbate; Q_e_ and C_e_ are the equilibrium concentrations of MB (g kg^−1^) and in the solution (g L^−1^).

### Adsorption kinetic studies

The effect of contact time on MB adsorption was investigated through kinetic studies. In each experiment, 25 mg of biochar was added to 10 mL of MB solution with an initial concentration of 300 mg L⁻^1^. The suspensions were agitated for time intervals ranging from 1 to 24 h. After each interval, the mixtures were centrifuged, and the residual MB concentration was measured spectrophotometrically at 664 nm. The pseudo-first-order rate constant k_1_ and equilibrium capacity q_e_ were determined from a plot of ln (q_e_-q_t_) versus t. The pseudo-second-order model provided k_2_ and q_e_ from a plot of t/q_t_ versus t. Intraparticle diffusion parameters k_id_ and C were obtained from a plot of q_t_ versus t^0.5^, identifying the contribution of boundary layer effects and intraparticle transport. The applicability of each isotherm model was evaluated by comparing the correlation coefficients (R^2^) obtained from the linear plots.

**Table:** 

Kinetic model	Linear form	plots	Refs.
Lagergren first order	$\ln \left( {{\mathrm{q}}_{{\mathrm{e}}} - {\mathrm{q}}_{{\mathrm{t}}} } \right) = \ln {\mathrm{q}}_{{\mathrm{e}}} - {\mathrm{K}}_{1} {\mathrm{t}}$	$\ln \left( {q_{e} - q_{t} } \right)$ versus t	^47^
pseudo-second order	$\frac{{\mathrm{t}}}{{{\mathrm{q}}_{{\mathrm{t}}} }} = { }\left[ {\frac{1}{{{\mathrm{K}}_{2} {\mathrm{q}}_{{\mathrm{e}}}^{2} }}} \right] + \frac{1}{{q_{e} }} t$	t/q_e_ versus t	^48^
Intraparticle diffusion	${\mathrm{q}}_{{\mathrm{t}}} = {\text{ K}}_{{\text{id }}} {\text{ t}}^{\frac{1}{2}} + {\mathrm{C}}$	q_t_ versus t^1/2^	^49^

Where q_e_ and q_t_ are the amount of dye adsorbed per unit mass of the adsorbent (in mg/g) at equilibrium time and time t, respectively, k is the rate constant, and C is the intraparticle diffusion constant.

### Elemental analysis

The elemental composition of the modified biochars, including carbon (C), hydrogen (H), nitrogen (N), and oxygen (O), was analyzed using a CHNS Vario EL III elemental analyzer (Elementar, Germany).

### FTIR analysis

The surface functional groups of pristine and modified biochars were analyzed using Fourier-transform infrared spectroscopy (FTIR, Bruker Vertex 80, Germany) with a spectral resolution of 4 cm⁻^1^ over the range of 4000–400 cm⁻^1^. For sample preparation, finely ground biochar was homogenized with spectroscopic-grade KBr, and pellets were pressed prior to measurement.

### SEM analysis

The morphological features of pristine and modified biochars were examined using scanning electron microscopy (SEM, Quanta FEG 250). Prior to imaging, the samples were sputter-coated with a thin layer of gold to improve conductivity. Micrographs were obtained at an accelerating voltage of 20 kV.

## Results and discussion

### Chemical constituent’s analyses of precursors

The suitability of agricultural residues for biochar production is strongly influenced by their chemical composition, particularly the proportions of cellulose, hemicellulose, lignin, extractives, and ash, as these components determine thermal behavior, carbon retention, and structural stability during pyrolysis. Among the feedstocks studied, giant reed (GR) exhibited the highest holocellulose content (83.3%), predominantly composed of α-cellulose, which provides structural uniformity, rigidity, and relatively high carbon content. However, its low lignin content (11.8%) and minimal ash (2.6%) limit fixed carbon retention during pyrolysis, resulting in a relatively low biochar yield (46.7%). Lignin, being more thermally stable than cellulose and hemicellulose, decomposes more slowly and contributes significantly to fixed carbon formation, thus enhancing overall char production and stability^50^. Date palm fiber (DP) displayed a more balanced chemical profile, with moderate holocellulose (69.9%), higher lignin (18.6%), and ash content (5.8%), which collectively support better thermal stability and char formation compared with GR. Nevertheless, the relatively high extractives content (15.8%) in DP may volatilize during pyrolysis, potentially reducing char stability and modifying surface chemistry. Rice straw (RS) emerged as the most promising precursor for high-performance biochar, containing the highest lignin fraction (20.4%) and ash content (15.9%), which favor thermal stability, fixed carbon retention, and catalyzed carbonization through mineral-assisted reactions. Additionally, the moderate α-cellulose content (43.6%) in RS provides sufficient structural integrity, enabling the formation of robust and porous biochar matrices suitable for adsorption applications. In contrast, sugarcane bagasse (SCB) appeared to be the least suitable feedstock for biochar production due to its high hemicellulose content (26.5%), which decomposes rapidly into volatiles, coupled with moderate lignin (19.1%) and low ash (4.7%). This composition results in reduced fixed carbon yield and weaker char stability, limiting its effectiveness as a high-quality biochar precursor^51^. Based on its chemical composition and predicted pyrolytic behavior, RS is likely to produce biochar of the highest quality, featuring superior carbon retention, improved surface functionality, and excellent adsorption potential. Conversely, SCB is expected to have the lowest capability for generating stable and effective biochar (Table 1).

**Table 1:** Chemical constituents of lignocellulosic biomass fibers.

Code	Extractive, %	Lignin, %	Holo-Cellulose, %	α-Cellulose, %	Hemi-Cellulose, %	Ash, %
GR	2.360	11.76	83.33	67.74	15.59	2.604
DP	15.76	18.55	69.96	38.99	24.79	5.820
SCB	7.320	19.10	68.90	41.60	26.50	4.700
RS	8.380	20.44	56.64	43.61	13.02	15.89

### Characterization of biochar and surface-modified biochar

#### Methylene blue adsorption performance

**Fig. 1:**
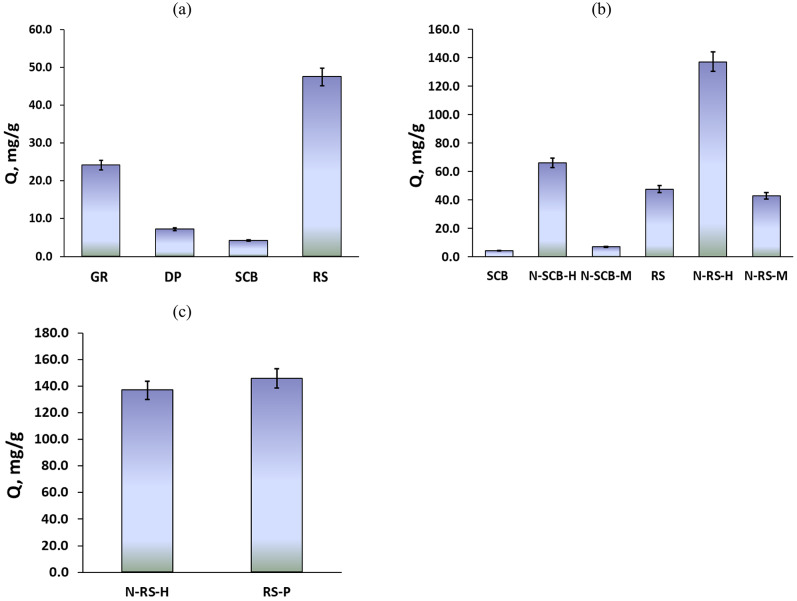
MB adsorption capacities of biochar form (**a**) different agricultural residues, (**b**) nitrogen doping on both RS and SCB via hydrothermal and microwave technique, (**c**) Hydrothermal N-doping and phosphoric acid activation of rice straw biochar.

The adsorption performance of biochar samples derived from different agricultural residues exhibited substantial variation, as illustrated in Fig. 1a. Rice straw (RS) biochar demonstrated the highest adsorption capacity, reaching 47.4 ± 2.4 mg/g. This superior performance is attributed to its high lignin and ash contents, which enhance fixed carbon yield, promote mineral-catalyzed carbonization, and facilitate the development of a porous structure rich in active sites during pyrolysis^52^. Consequently, RS biochar was considered the most efficient adsorbent among the tested feedstocks. Giant reed (GR) biochar showed a moderate adsorption capacity of 24.1 ± 1.2 mg/g. Although GR is rich in holocellulose and α-cellulose, its relatively low lignin and ash fractions limit the stability of carbon structures and the extent of active site formation. Nevertheless, GR biochar outperformed both date palm fiber (DP) and sugarcane bagasse (SCB) biochar, highlighting that holocellulose-rich residues can provide moderate adsorption performance when lignin content is insufficient for high char stability.

In contrast, DP and SCB biochars exhibited much lower adsorption capacities (7.2 ± 0.4 and 4.2 ± 0.2 mg/g, respectively). SCB’s poor performance is primarily due to its high hemicellulose content, which decomposes rapidly into volatiles rather than contributing to stable carbon structures. DP biochar, although richer in lignin and ash than SCB, contains a high extractives fraction (15.8%), which can interfere with pore development and reduce surface functional group availability. Overall, the adsorption capacity followed the trend: RS > GR > DP > SCB, underscoring the critical role of feedstock composition in determining biochar performance. To enhance adsorption capacity, nitrogen (N) doping was applied to both RS and SCB biochar (Fig. 1b). This approach allowed evaluation of the potential of surface modification to compensate for intrinsic compositional limitations. Nitrogen doping significantly improved adsorption, with hydrothermal treatment (H) outperforming microwave treatment (M). For SCB, hydrothermal N-doping (N-SCB-H) increased adsorption capacity to 65.9 ± 3.3 mg/g, over tenfold higher than pristine SCB (4.2 ± 0.2 mg/g), whereas N-SCB-M achieved only a marginal improvement (7.2 ± 0.4 mg/g). These results indicate that hydrothermal N-doping effectively introduces nitrogen functional groups and active sites to hemicellulose-rich biomass, while microwave treatment is insufficient for SCB.

For RS, nitrogen modification further amplified adsorption efficiency. Hydrothermally N-doped RS (N-RS-H) achieved the highest adsorption capacity of 136.9 ± 6.9 mg/g, nearly three times that of pristine RS. This substantial improvement highlights the synergistic effect of an intrinsically favorable feedstock composition and targeted nitrogen functionalization. In contrast, N-RS-M displayed intermediate performance (42.7 ± 2.1 mg/g), comparable to pristine RS, suggesting that microwave doping did not adequately modify surface chemistry or porosity. Notably, the adsorption capacity of N-RS-H (136.9 ± 6.9 mg/g) was comparable to that of phosphoric-acid activated RS (RS-P, 145.7 ± 7.3 mg/g) (Fig. 1c). While phosphoric acid activation effectively creates high surface area and extensive porosity^53^, it requires concentrated acid, generates phosphate-rich effluents, and produces large volumes of wastewater during neutralization and washing^54^. Hydrothermal N-doping, on the other hand, introduces pyridinic, pyrrolic, and graphitic nitrogen groups^55^, enhancing surface chemistry with minimal hazardous byproducts and allowing easier recycling of process water. These findings suggest that hydrothermal N-doping can achieve adsorption efficiencies comparable to chemical activation while minimizing environmental impact, representing a more sustainable modification pathway.

The adsorption of methylene blue (MB) onto biochar and their modified forms was evaluated using Langmuir, Freundlich, Temkin, and Dubinin–Radushkevich (D–R) isotherm models. The Langmuir model provided the best fit (R^2^ = 0.97–1.00), confirming monolayer adsorption on homogeneous surfaces. The dimensionless separation factor (R_L_ = 0.005–0.236) indicated favorable adsorption. The binding affinity constant (b) was highest for RS-P (0.363 L mg⁻^1^), reflecting enhanced interactions between MB and oxygenated surface sites. Enhanced adsorption can be attributed to increased porosity and surface functionalities introduced by activation, facilitating electrostatic interactions with cationic MB. Temkin analysis revealed strong adsorbate–adsorbent interactions for DP and SCB, although overall adsorption remained limited. D–R modeling yielded mean adsorption energies of 0.04–0.75 kJ mol⁻^1^, suggesting physical adsorption mechanisms dominated, including electrostatic and van der Waals forces. These findings collectively highlight that the composition of the feedstock and the strategy for its modification are crucial factors influencing adsorption performance. Among the various options, biochar derived from RS, especially those that are hydrothermally N-doped and activated with phosphoric acid, stand out as the most effective adsorbents for treating dye-polluted wastewater (Table 2).

**Table 2:** Langmuir, Freundlich, Temkin and dubinin-radushkevich (D–R) isotherm parameters for adsorption of MB dye onto all investigated biochar prepared different biowastes and their modifications.

Model name	Isotherm parameters	GR	DP	SCB	N-SCB-H	N-SCB-M	RS	N-RS-H	N-RS-M	RS-P
Langmuir isotherm	Q_m_, mg g^−1^	24.125	7.176	4.203	65.920	6.886	47.438	136.986	42.662	145.773
	b, L mg^−1^	0.005	0.175	0.123	0.031	0.010	0.155	0.114	0.112	0.363
	R^2^	0.999	1.000	0.997	0.974	0.970	1.000	0.994	0.997	1.000
	R_L_, mg L^−1^	0.236	0.009	0.013	0.051	0.149	0.011	0.014	0.015	0.005
Freundlich isotherm	1/n	0.878	1.000	0.068	0.391	1.731	0.274	1.676	5.844	2.889
	K_F_, mg g^−1^	5.362	1.000	6.368	8.076	14.028	13.684	108.693	6.95E + 06	4.36E + 04
	R^2^	0.976	1.000	0.684	0.807	0.889	0.946	0.945	0.690	0.658
Temkin isotherm	B, J.mol^−1^	0.503	4.160	8.025	0.163	1.540	0.310	0.085	0.403	0.124
	K_T_, L.mg^−1^	17.462	3.69E + 07	2.81E + 08	3.973	12.510	2.899	1.191	4.621	14.610
	R^2^	0.991	0.998	0.684	0.824	0.949	0.985	0.983	0.689	0.785
D-R isotherm	β, mol^2^ KJ^−2^	2.00E-04	2.00E-05	2.00E-05	1.00E-04	3.00E-04	2.00E-06	9.00E-07	1.00E-05	1.00E-06
	Q_m_, mg g^−1^	10.750	7.566	4.562	56.895	4.831	39.536	79.639	42.191	142.494
	E_D-R_, KJ mol^−1^	0.050	0.158	0.158	0.071	0.041	0.500	0.745	0.224	0.707
	R^2^	0.914	0.827	0.338	0.993	0.988	0.928	0.850	0.939	0.970

### Specific surface area

The specific surface area of biochar plays a crucial role in determining their adsorption performance, as it directly correlates with the availability of active sites for contaminant binding. In this study, the Langmuir isotherm method was employed to estimate the specific surface area (S_MB_) based on the monolayer adsorption capacity (Q_m_) of methylene blue (MB). Following the approach reported by Itodo et al.^44^, Langmuir type I is appropriate for evaluating S_MB_ because it assumes monolayer coverage on a homogeneous surface, which aligns well with the adsorption characteristics of biochar materials. The Q_m_ values obtained from Langmuir fitting were subsequently used to calculate the S_MB_ of each biochar sample. The results, summarized in Table 3, demonstrate clear differences in specific surface area among the tested biochar, reflecting the combined influence of feedstock composition and modification strategy. Among the pristine biochar, rice straw (RS) exhibited the highest specific surface area (150.6 × 10⁻^3^ km^2^ kg⁻^1^), consistent with its superior Q_m_ value (47.4 mg/g). This high surface area can be attributed to its balanced chemical composition, including relatively high lignin and ash contents, which favor the formation of a porous and stable carbon matrix during pyrolysis. In comparison, giant reed (GR, 76.6 × 10⁻^3^ km^2^ kg⁻^1^), date palm fiber (DP, 22.8 × 10⁻^3^ km^2^ kg⁻^1^), and sugarcane bagasse (SCB, 13.3 × 10⁻^3^ km^2^ kg⁻^1^) displayed significantly lower surface areas, consistent with their lower adsorption capacities. The lower porosity of SCB and DP can be explained by their high hemicellulose and extractives content, which volatilize during pyrolysis and limit the development of stable pores.

**Table 3:** Specific surface area of all investigated biochar prepared different biowastes and their modifications.

	Q_m_	S_MB_ (10^-3^km^2^kg^-1^)
GR	24.13 ± 1.21	76.599
DP	7.18 ± 0.36	22.783
SCB	4.20 ± 0.21	13.346
N-SCB-H	65.92 ± 3.29	209.297
N-SCB-M	6.89 ± 0.34	21.864
RS	47.44 ± 2.37	150.618
N-RS-H	136.99 ± 6.85	434.936
N-RS-M	42.66 ± 2.13	135.454
RS-P	145.77 ± 7.29	462.832

Chemical modification of biochar significantly enhanced their specific surface area. Hydrothermally nitrogen-doped RS (N-RS-H) and phosphoric acid-activated RS (RS-P) achieved the highest S_MB_ values of 434.9 × 10⁻^3^ and 462.8 × 10⁻^3^ km^2^ kg⁻^1^, respectively, with corresponding adsorption capacities of 136.9 ± 6.9 and 145.7 ± 7.3 mg/g. These improvements reflect the synergistic effect of pore development and the introduction of surface functional groups. Phosphoric acid activation promotes the formation of a sponge-like porous network enriched with oxygenated and phosphate groups, while hydrothermal nitrogen doping introduces pyridinic, pyrrolic, and graphitic N functionalities that increase adsorption sites. In contrast, microwave nitrogen doping of RS (N-RS-M) yielded only a moderate surface area enhancement (135.5 × 10⁻^3^ km^2^ kg⁻^1^), highlighting the importance of the modification method in determining porosity and surface chemistry. A similar trend was observed for SCB biochar. Hydrothermally nitrogen-doped SCB (N-SCB-H) exhibited a marked increase in specific surface area (209.3 × 10⁻^3^ km^2^ kg⁻^1^) compared with pristine SCB (13.3 × 10⁻^3^ km^2^ kg⁻^1^) and microwave-treated SCB (N-SCB-M, 21.9 × 10⁻^3^ km^2^ kg⁻^1^). This dramatic improvement demonstrates that hydrothermal nitrogen doping is highly effective in generating porosity and functional sites, even in hemicellulose-rich, low-lignin biomass that is inherently less suitable for biochar production. The findings suggest that the composition of the feedstock and the selected modification method significantly affect the specific surface area of biochar. Techniques like hydrothermal nitrogen doping and phosphoric acid activation greatly enhance surface area and porosity, leading to improved adsorption capabilities. In contrast, less intensive methods, such as microwave doping, result in only minor enhancements. These results highlight the necessity of customizing modification approaches to align with the inherent characteristics of the biomass precursor to optimize biochar-based adsorption applications.

### Iodine value

**Fig. 2:**
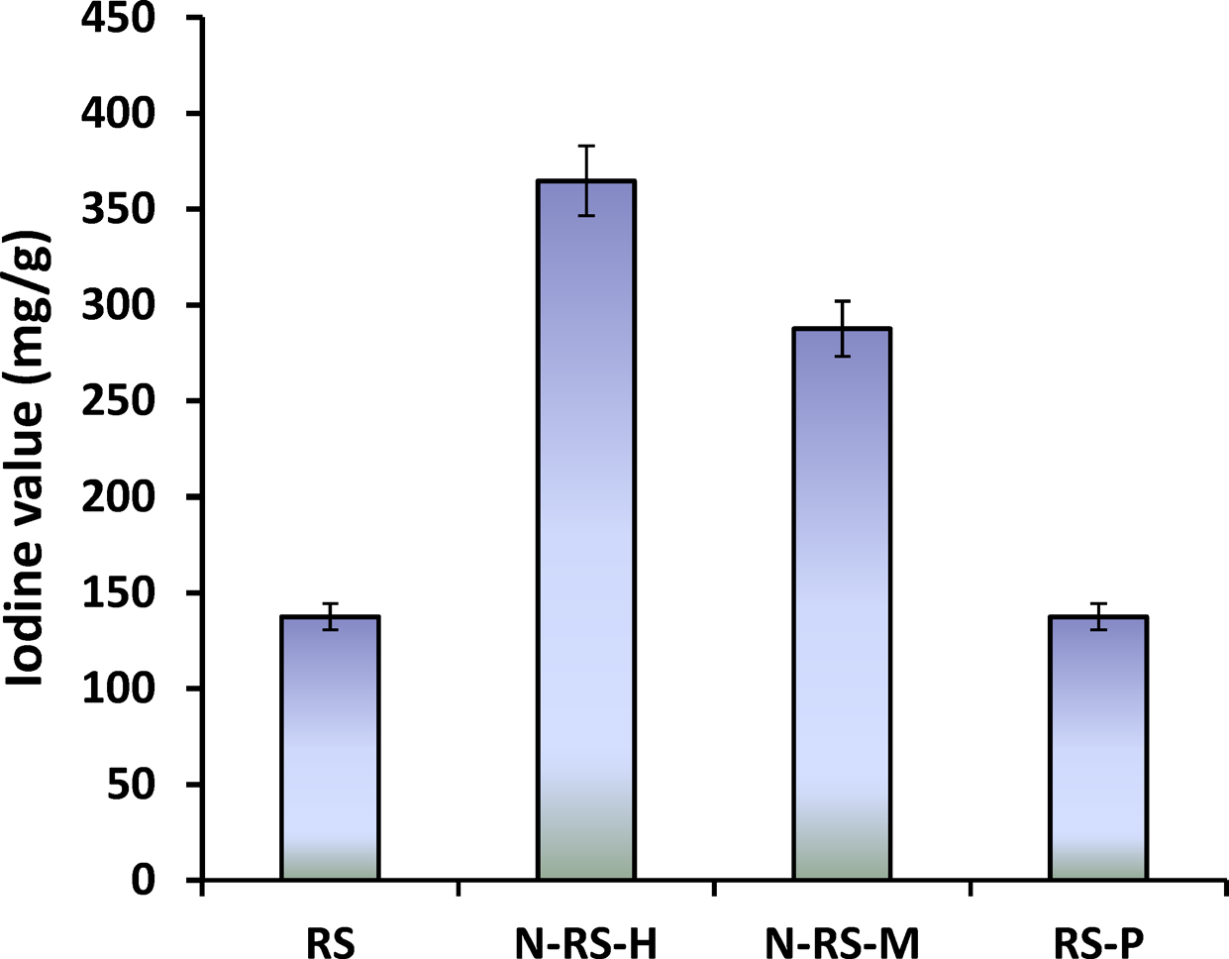
Iodine values of the samples based on RS biochar.

Based on the obtained data, rice straw (RS) biochar and its modifications via hydrothermal nitrogen doping (N-RS-H), microwave nitrogen doping (N-RS-M), or phosphoric acid activation (RS-P) demonstrated superior methylene blue (MB) adsorption performance, warranting their selection for further in-depth evaluation. One of the key indicators of biochar adsorption potential is the iodine number, a widely accepted parameter for assessing micropore availability and overall adsorption capacity. The iodine number reflects the volume of micropores (0–20 Å) accessible to small molecules in aqueous solutions, providing insights into the structural porosity that contributes to pollutant uptake. As illustrated in Fig. 2, nitrogen-doped RS biochar exhibited substantially higher iodine numbers compared with both pristine RS and phosphoric acid-activated RS-P. Hydrothermally modified N-RS-H achieved the highest iodine value of 364.8 ± 18.2 mg/g, followed by N-RS-M at 287 ± 14.4 mg/g, whereas unmodified RS and RS-P displayed significantly lower values of 137 ± 6.9 mg/g. These results clearly indicate that nitrogen doping, particularly under hydrothermal conditions, effectively enhances microporosity and increases specific surface area, thereby creating more accessible adsorption sites for MB molecules. The hydrothermal route likely facilitates deeper incorporation of nitrogen functional groups (e.g., pyridinic, pyrrolic, and graphitic N), which promote crosslinking within the carbon matrix and prevent pore collapse, resulting in a more robust microporous network^56^.

Interestingly, RS-P, despite exhibiting strong MB adsorption in isotherm studies, showed an iodine number comparable to unmodified RS. This suggests that its enhanced adsorption capacity arises primarily from surface chemical functionalization, such as hydroxyl (–OH), phosphate (–PO₄), and carboxyl (–COOH) groups, rather than an increase in micropore volume^57^. These functional groups provide sites for electrostatic interactions, hydrogen bonding, and π–π stacking with MB molecules, demonstrating that surface chemistry can compensate for limited microporosity in adsorption performance. By contrast, the elevated iodine values observed for N-RS-H and N-RS-M confirm that nitrogen incorporation predominantly promotes the formation of a microporous structure and increases the density of active sites, thereby strengthening overall adsorption efficiency. Moreover, the comparison between hydrothermal and microwave doping highlights the critical role of the modification method: hydrothermal treatment achieves deeper nitrogen integration, higher microporosity, and greater surface area, whereas microwave doping produces more moderate enhancements. These findings collectively emphasize the synergistic roles of microporosity and surface functionalization in shaping the adsorption efficiency of biochar, with nitrogen doping identified as a potent method to enhance both its structural and chemical characteristics at the same time.

### Impact of functionalization of RS-biochar on its characterization

#### Surface chemistry (acidic and basic groups)

The Boehm titration analysis (Table 4) highlights pronounced variations in the surface chemistry of rice straw (RS) biochar and its modified derivatives, emphasizing the impact of modification strategies on functional group distribution. Pristine RS exhibited a predominance of acidic functional groups (4.122 mmol g^−1^) over basic sites (0.824 mmol g^−1^), reflecting its inherently acidic surface chemistry. This composition favors electrostatic attraction toward cationic adsorbates such as methylene blue (MB), consistent with previous observations on lignocellulosic biochar where naturally occurring oxygenated groups (–COOH, –OH, –C=O) play a key role in cation binding. Nitrogen doping, implemented through hydrothermal (N-RS-H) and microwave-assisted (N-RS-M) methods, altered this acid–base balance. N-RS-H displayed a slight decrease in acidic groups (3.819 mmol g^−1^) accompanied by a substantial increase in basic sites (1.495 mmol g^−1^), whereas N-RS-M showed a moderate reduction in acidity (3.627 mmol g^−1^) alongside a similar increase in basic functionalities (1.487 mmol g^−1^). These modifications indicate that nitrogen incorporation introduces electron-rich sites such as pyridinic, pyrrolic, and graphitic nitrogen, which enhance π–π interactions and electron-donor effects during dye adsorption. The hydrothermal method appears particularly effective in promoting uniform nitrogen incorporation throughout the carbon matrix, improving both active site density and accessibility.

**Table 4:** Acidic and basic functional groups on the surface of samples based on RS biochar.

Biochar code	Acidic groups	Basic groups	References
	mmol g^-1^	mmol g^-1^	
RS	4.122	0.824	Present study
N-RS-H	3.819	1.495	
N-RS-M	3.627	1.487	
RS-P	8.276	0.332	
Peach stones	1.300	0.820	^58^
Activated carbons (ACs) obtained from peach stones with phosphoric acid activation at 400 °C	1.550	0.750	
ACs obtained from peach stones with with phosphoric acid activation at 400 °C followed by 45% HNO_3_ modification at 90 °C for 2 h	7.700	0.450	
ACs from bean husk carbonization at 350 °C for 2 h followed by activation with 0.3 M phosphoric acid	1.397	0.700	^59^
ACs from kola nut pod carbonization at 350 °C for 2 h followed by activation with 0.3 M phosphoric acid	0.136	0.038	
ACs from coconut husk carbonization at 350 °C for 2 h followed by activation with 0.3 M phosphoric acid	2.034	0.096	
ACs from wheat bran activation (CO_2_ or/and steam)	1.949	1.370	^60^
Eucalyptus wood biochars produced at pyrolysis temperatures of 450 C with acid–base treatment	0.630	0.630	Tsechansky & Graber^61^
Eucalyptus wood biochars produced at pyrolysis temperatures of 600 C with acid–base treatment	0.340	0.320	

In contrast, phosphoric acid activation (RS-P) produced a markedly acidity-dominant surface, with the highest concentration of acidic groups (8.276 mmol g^−1^) and a simultaneous suppression of basic sites (0.332 mmol g^−1^). This pronounced enrichment in acidic functionalities aligns with earlier studies on phosphoric acid–activated carbons derived from peach stones, where acid treatment promoted oxygenated moieties (carboxylic, phenolic, lactonic) at the expense of basic groups ^58^. Comparatively, RS-P exhibited substantially higher acidic group densities than biochar from other agricultural residues, including bean husk (1.397 mmol g^−1^), coconut husk (2.034 mmol g^−1^), and wheat bran (1.949 mmol g^−1^)^59,60^, confirming the efficacy of phosphoric acid activation in generating oxygen-containing acidic moieties. Conversely, eucalyptus wood biochar reported by Tsechansky and Graber^61^ contained significantly lower concentrations of both acidic and basic groups (< 1 mmol g^−1^), particularly when subjected to high pyrolysis temperatures, highlighting the thermal degradation of surface functionalities.

All these results emphasize the potential to fine-tune surface chemistry for improved adsorption performance. RS-P is characterized by a surface with predominant acidity, enhancing electrostatic interactions with cationic dyes. Conversely, N-RS-H and N-RS-M exhibit a more balanced acid–base chemistry, enabling both electrostatic and π–π interactions. Mechanistically, acidic groups such as –COOH, –OH, and –C = O, along with phosphate functionalities, are primarily responsible for cationic dye adsorption through electrostatic attraction. In contrast, basic nitrogen groups like –NH₂, pyridinic N, and graphitic N, along with conjugated carbon domains, facilitate π–π stacking with the aromatic rings of MB. The combination of these mechanisms’ accounts for the superior adsorption efficiency observed in nitrogen-doped and phosphoric acid–activated RS biochar, underscoring the importance of tailoring surface functionalization to the chemical composition of the feedstock.

### Adsorption batch experiment

**Fig. 3:**
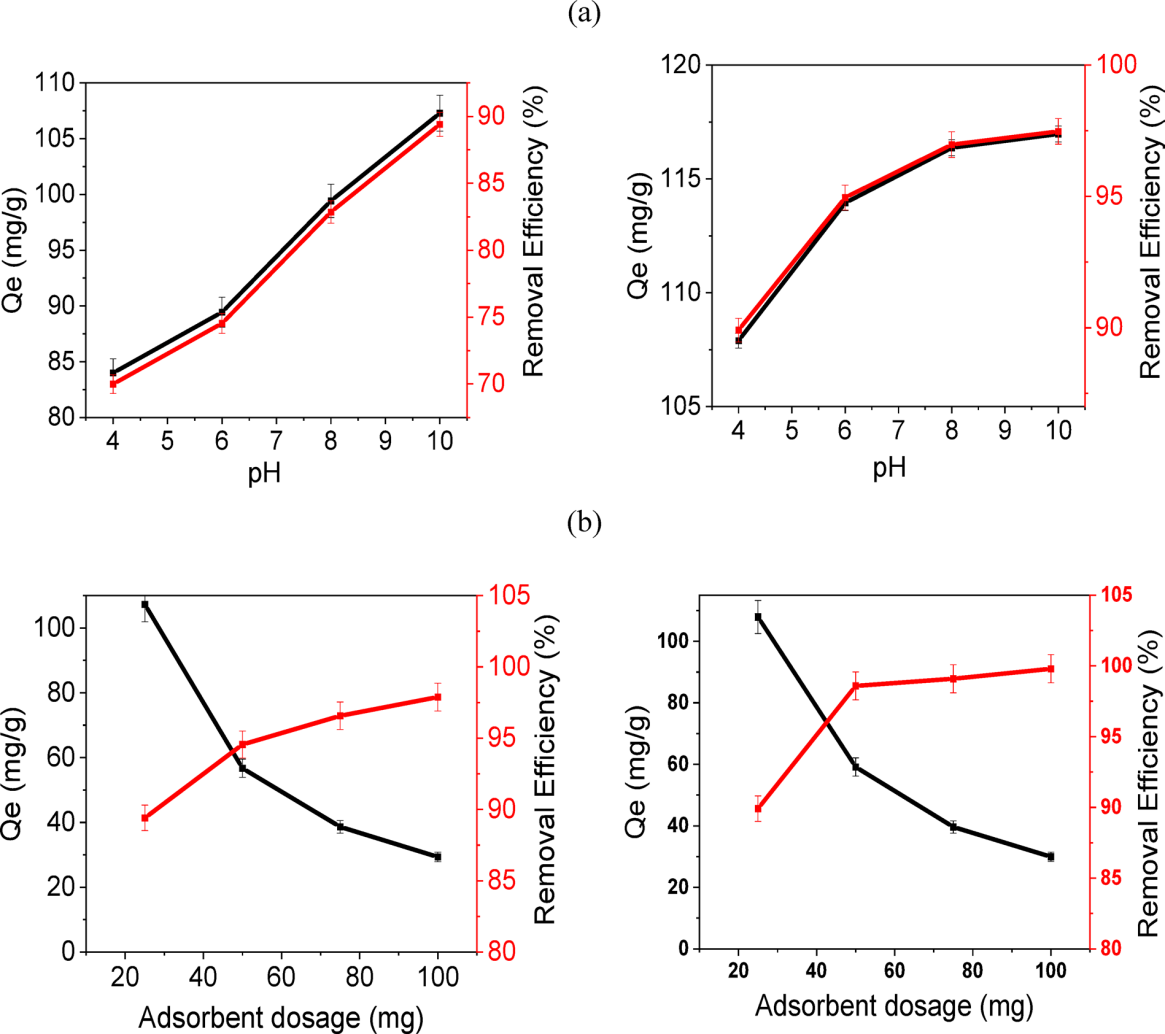
The adsorption capacity and the removal efficiency of MB by N-RS-H and RS-P: (**a**) pH and (**b**) adsorbent dosage.

**Fig. 4:**
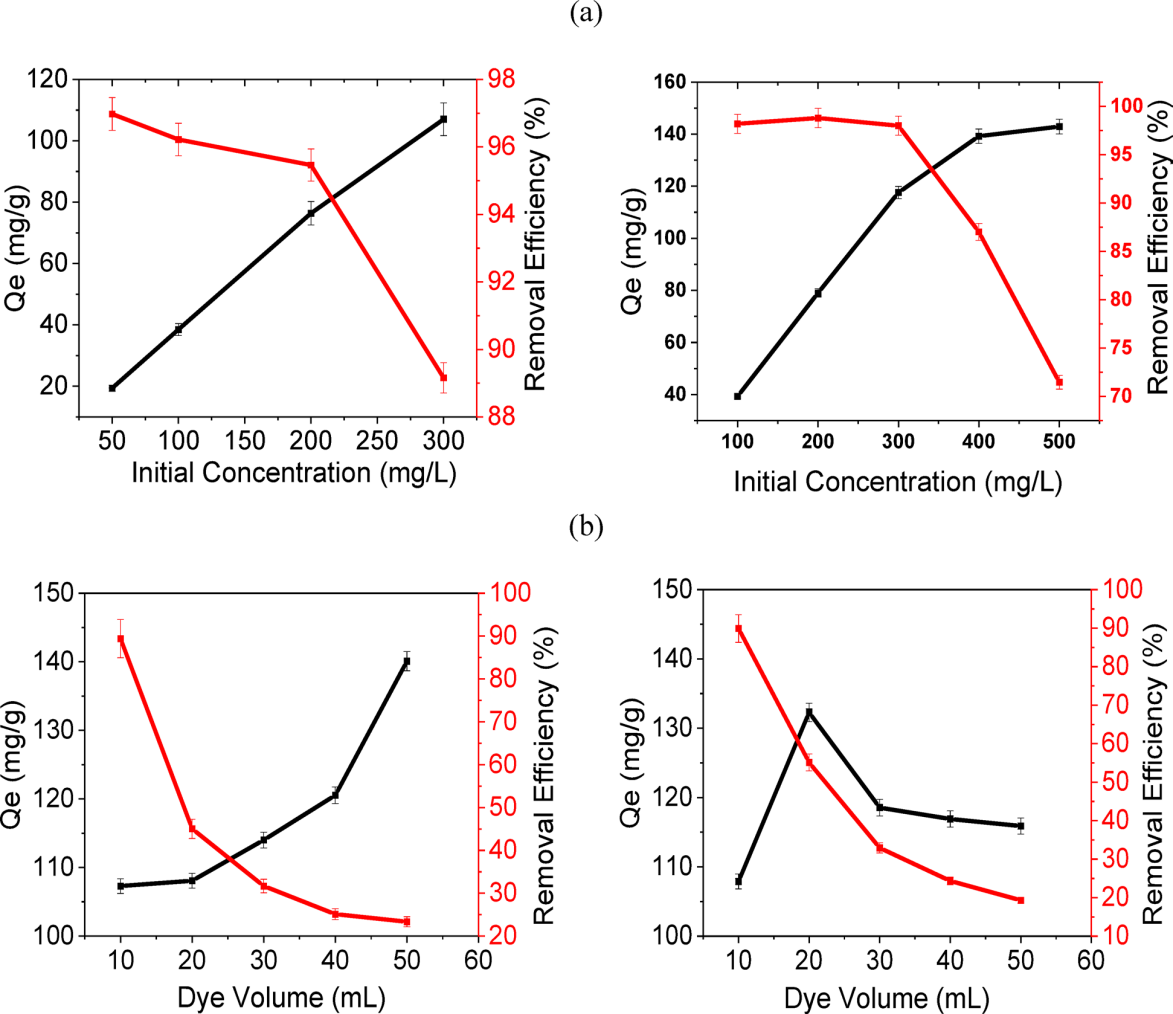
The adsorption capacity and the removal efficiency of MB by N-RS-H and RS-P: (**a**) initial concentration and (**b**) dye volume.

Rice straw–derived biochar modified via hydrothermal nitrogen doping (N-RS-H) and phosphoric acid activation (RS-P) exhibited superior adsorption performance toward methylene blue (MB) and were therefore selected for detailed batch adsorption experiments, as illustrated in Figs. 3 and 4. The experiments revealed that adsorption capacity (Qe) and removal efficiency (%) were strongly influenced by solution pH, adsorbent dosage, initial dye concentration, and dye volume, highlighting the interplay between surface chemistry, electrostatics, and adsorbate availability.

### Effect of pH

The adsorption behavior of N-RS-H was markedly pH-dependent. Both adsorption capacity and removal efficiency increased progressively as pH rose, from 83.9 ± 0.8 mg/g at pH 4 to 107 ± 1.1 mg/g at pH 10, while removal efficiency improved from 69 ± 0.7 to 89.4 ± 0.9% (Fig. 3a). This trend reflects the surface charge dynamics of biochar in aqueous solutions. Under acidic conditions, the biochar surface is protonated, leading to partial electrostatic repulsion between positively charged surface sites and the cationic MB molecules, thereby limiting adsorption. As the solution becomes more alkaline, deprotonation of acidic functional groups (–COOH, –OH, –C=O) increases the net negative surface charge, strengthening electrostatic attraction toward MB cations. Such pH-dependent adsorption behavior has been consistently reported in other lignocellulosic and nitrogen-doped biochar^55,58^, confirming the critical role of functional group speciation. In contrast, RS-P exhibited consistently higher adsorption capacities across the entire pH range (107.8 ± 0.54 to 116.9 ± 0.6 mg g^−1^) and achieved removal efficiencies above 90%, even under moderately acidic conditions. The exceptional performance of RS-P can be attributed to its high density of acidic surface groups (8.276 mmol g^−1^), which provide abundant negatively charged sites for electrostatic interactions with MB molecules, regardless of the surrounding pH. The relatively flat adsorption profile at higher pH values indicates that the biochar surface reaches saturation, where the available binding sites are fully occupied and further increases in electrostatic potential do not significantly enhance adsorption. These observations highlight that both the nature and density of surface functional groups, in combination with solution pH, govern the adsorption efficiency of biochar. Nitrogen doping introduces basic sites and electron-rich functionalities that complement acidic groups, promoting additional interactions such as π–π stacking and hydrogen bonding. Phosphoric acid activation, on the other hand, maximizes acidic site density and maintains high negative surface charge under a wide pH range, thereby providing consistently strong adsorption through electrostatic attraction. The pH-dependent adsorption behavior emphasizes the importance of tailoring biochar surface chemistry to target specific cationic pollutants for optimal removal efficiency.

### Effect of adsorbent dosage

The influence of adsorbent dosage on methylene blue (MB) removal was evaluated to understand the relationship between the number of available binding sites and adsorption efficiency. Increasing the adsorbent dosage improved the removal efficiency for both N-RS-H and RS-P biochar, reflecting the greater availability of surface sites for dye interaction. For N-RS-H, the adsorption capacity (Qe) decreased from 107.3 ± 1.1 to 29.4 ± 0.88 mg g^−1^as the dosage increased from 25 to 100 mg, while removal efficiency increased from 89.4 ± 1.7 to 97.9 ± 1.5% (Fig. 3b). This inverse trend is typical in adsorption studies and is attributed to the reduced adsorbate-to-adsorbent ratio at higher dosages: although more sites are available overall, each unit mass of adsorbent captures less dye. For RS-P, a similar inverse relationship was observed, with Qe declining more sharply from 107.9 ± 1.1 to 29.9 ± 0.3 mg g^−1^ as dosage increased. However, removal efficiency reached nearly 100% at dosages of 50–100 mg, indicating that RS-P achieves complete MB removal with a smaller amount of adsorbent. This behavior is consistent with its high density of acidic surface groups (8.276 mmol g^−1^), which provide abundant negatively charged sites to promote strong electrostatic interactions with cationic MB molecules^58^. These results highlight that RS-P is more material-efficient than N-RS-H for achieving maximum dye removal.

### Effect of initial dye concentration

The effect of varying MB concentration on adsorption capacity and removal efficiency reflects the capacity of the biochar to accommodate increasing solute loads. For N-RS-H, Qe increased from 19.4 ± 0.99 mg g^−1^ at 50 mg/L to 107.3 ± 1.1 mg g^−1^ at 300 mg L^−1^, while removal efficiency remained relatively stable at lower concentrations (96.9 ± 0.5% at 50 mg/L to 95.5 ± 4.5% at 200 mg L^−1^) before declining to 89.4 ± 1.7% at 300 mg L^−1^ (Fig. 4a). The reduction in removal efficiency at higher concentrations indicates partial saturation of available active sites, where the number of MB molecules exceeds the adsorption capacity of the biochar surface.RS-P consistently exhibited higher Qe values across the tested range (39.3 ± 0.8–142.9 ± 2.8 mg g^−1^ for 100–500 mg L^−1^), demonstrating superior affinity toward MB due to its dense population of acidic functional groups that facilitate electrostatic binding. Nevertheless, removal efficiency decreased markedly beyond 300 mg L^−1^, reaching 71.5 ± 0.8% at 500 mg L^-1^, suggesting that even the enhanced surface functionalities of RS-P have a finite capacity for MB, beyond which dye molecules remain unadsorbed. The adsorption isotherm data were well described by the Langmuir model, with high correlation coefficients (R^2^ = 0.994–0.999), indicating monolayer adsorption on a homogeneous surface. The maximum adsorption capacities were determined to be 136.99 ± 6.85 mg g⁻^1^ for N-RS-H and 145.77 ± 7.29 mg g⁻^1^ for RS-P. Table 5 presents a comparative summary of methylene blue (MB) adsorption capacities of biochars derived from various agro-wastes under different experimental conditions. As shown in Table 5, the adsorption capacities obtained in this study are comparable to or higher than those reported for other biochar-based adsorbents.

**Table 5:** Comparison of Langmuir MB adsorption of our case study versus literature based various agro-wastes biochar.

Biochar/Material	MB adsorption capacity (qₘ)	References
Biochar microparticles from pine wood, pig manure, cardboard	7.8–25 mg g^-1^	^62^
Ball-milled rice straw biochar	~ 50.27 mg g^-1^	^63^
Activated bamboo biochar (KBBC-900)	~ 67.46 mg g^-1^	^64^
Jackfruit peel biochar	~ 39.87 mg g^-1^	^65^
Natural biochar (Biochar A)	~ 86.95 mg g^-1^	^66^ )
Kenaf fiber biochar	~ 164.21 mg g^-1^	^67^
Hydrothermally N-doped RS	136.99 ± 6.85	Present study
phosphoric acid activation RS	145.77 ± 7.29	Present study

### Effect of dye solution volume

The solution volume also impacted adsorption performance, reflecting changes in the adsorbent-to-adsorbate ratio. Increasing the volume from 10 to 50 mL led to reduced removal efficiencies for both biochar. For N-RS-H, Qe increased from 107.3 ± 1.1 to ~ 140.1 ± 1.5 mg g^-1^ due to a larger number of dye molecules being adsorbed per unit mass; however, removal efficiency dropped sharply from 89.4 ± 1.7 to 23.3%. RS-P exhibited a similar trend, with Qe rising from 107.9 ± 0.9 to 132.3 ± 1.5 mg g^-1^ before declining slightly to 115.8 ± 1.1 mg g^-1^, while removal efficiency decreased from 89.9 ± 3.5 to 19.3 ± 1.2% (Fig. 4b). These observations are consistent with reduced adsorbent-to-adsorbate ratios at higher volumes, which limit the proportion of MB removed despite the high intrinsic affinity of the biochar.

The adsorption performance of N-RS-H matched that of RS-P under different experimental conditions. The biochar showed pH-dependent characteristics because N-RS-H performance improved at higher pH levels through surface deprotonation, yet RS-P maintained high adsorption because of its acidic properties. The results showed that RS-P delivered better performance at low dosages and dye concentrations, but N-RS-H delivered matching results when pH and loading conditions were optimized. This proves that hydrothermal nitrogen doping improves surface chemistry and active site density. The two adsorbents, N-RS-H and RS-P, showed equal effectiveness for MB removal under optimal pH and dosage, and solute load conditions. This proves nitrogen-doped biochar functions at the same level as chemically activated biochar.

### Effect of temperature and thermodynamic parameters

Temperature plays a critical role in adsorption processes, influencing both the kinetics and the equilibrium capacity of the adsorbent. In this study, the thermodynamic behavior of methylene blue (MB) adsorption onto N-RS-H and RS-P biochar was assessed over a temperature range of 298–313 K at a constant initial MB concentration of 300 mg L^−1^, while maintaining other experimental parameters constant. The thermodynamic parameters Gibbs free energy (ΔG°), enthalpy (ΔH°), and entropy (ΔS°) were calculated following the equations reported in Basta et al.^39^, and the results are summarized in Table 6.

**Table 6:** Thermodynamic parameters for MB adsorption by both N-RS-H and RS-P.

Thermodynamic parameters	N-RS-H	RS-P
ΔH (KJ/mol)	51.352	41.544
ΔS (J/mol.K)	179.067	145.736
∆G (KJ/mol) (298)	− 1.907	− 1.503
∆G (KJ/mol) (303)	− 3.066	− 3.203
∆G (KJ/mol) (313)	− 4.639	− 3.860

The calculated parameters indicate that MB adsorption on both biochar is endothermic and spontaneous. The positive ΔH° values (51.35 kJ/mol for N-RS-H and 41.54 kJ/mol for RS-P) suggest that adsorption requires heat input, reflecting strong interactions between MB molecules and biochar surface sites, as well as possible pore-filling mechanisms. The higher ΔH° for N-RS-H implies that adsorption on nitrogen-doped biochar is more temperature-dependent, likely due to the activation of additional binding sites and enhanced accessibility of micropores at elevated temperatures. This behavior aligns with previous studies reporting that nitrogen functionalization can promote chemisorption or enhanced physisorption via electron-donating interaction. Positive ΔS° values (179.07 J/mol·K for N-RS-H and 145.74 J/mol·K for RS-P) indicate an increase in randomness at the solid–liquid interface during adsorption. This increase is commonly attributed to the displacement of water molecules from biochar surfaces and the reorganization of solvation shells surrounding the MB molecules. The larger ΔS observed for N-RS-H suggests greater structural reorganization and flexibility in accommodating MB molecules, likely due to the synergistic effects of nitrogen functionalities and porous architecture.

The negative ΔG° values across the temperature range (− 1.907 to − 4.639 kJ/mol for N-RS-H; − 1.503 to − 3.860 kJ/mol for RS-P) confirm the spontaneity of adsorption. Notably, ΔG° becomes more negative with increasing temperature, illustrating that the adsorption process is more thermodynamically favorable at higher temperatures. This trend corroborates the endothermic nature of MB adsorption and indicates that both electrostatic and π–π interactions between the biochar surface and MB molecules are strengthened at elevated temperatures. The thermodynamic analysis shows N-RS-H biochar demonstrates elevated enthalpic and entropic values compared to RS-P biochar which leads to increased temperature sensitivity and more complex adsorbate–adsorbent interaction changes. The dense acidic functionalities in RS-P create better spontaneous reactions at moderate temperatures which shows how chemical activation works together with surface functionalization to affect adsorption thermodynamics. The research findings show that biochar modifications which scientists create can improve adsorption at different temperatures to address wastewater treatment needs.

### Adsorption kinetic studies

The adsorption kinetics of methylene blue (MB) onto hydrothermally nitrogen-doped rice straw biochar (N-RS-H) and phosphoric acid-activated rice straw biochar (RS-P) were analyzed using the Lagergren first-order, pseudo-second-order, and intraparticle diffusion models. These models are commonly employed to characterize adsorption behavior from aqueous solutions and to elucidate the rate-limiting steps involved in the process. The Lagergren first-order model exhibited limited agreement with experimental data, as indicated by correlation coefficients (R^2^) of 0.955 for N-RS-H and 0.895 for RS-P (Table 7). Additionally, this model overestimated the equilibrium adsorption capacities (q_eq_ = 221–251 mg g⁻^1^), demonstrating that simple physisorption was not the dominant mechanism. This deviation is consistent with literature reports for dye adsorption on chemically modified biochar, where more complex surface interactions and functional group contributions are involved^68^.

**Table 7:** Kinetic parameters for MB adsorption by both N-RS-H and RS-P.

Kinetic parameters		N-RS-H	RS-P
Lagergren first order model	K_1_(h^−1^)	0.011	0.006
	q_eq_ (mg g^−1^)	251.464	221.030
	R^2^	0.955	0.895
	SEE	0.024	0.020
Pseudo-second order	K_2_ (mg g^−1^ h^−1^)	0.003	0.010
	q_eq_ (mg g^−1^)	119.332	111.607
	R^2^	0.992	0.999
	SEE	0.007	0.003
Intraparticle diffusion	K_id_	15.980	8.151
	C	30.113	69.228
	R^2^	0.985	0.952
	SEE	3.387	3.084

**Fig. 5:**
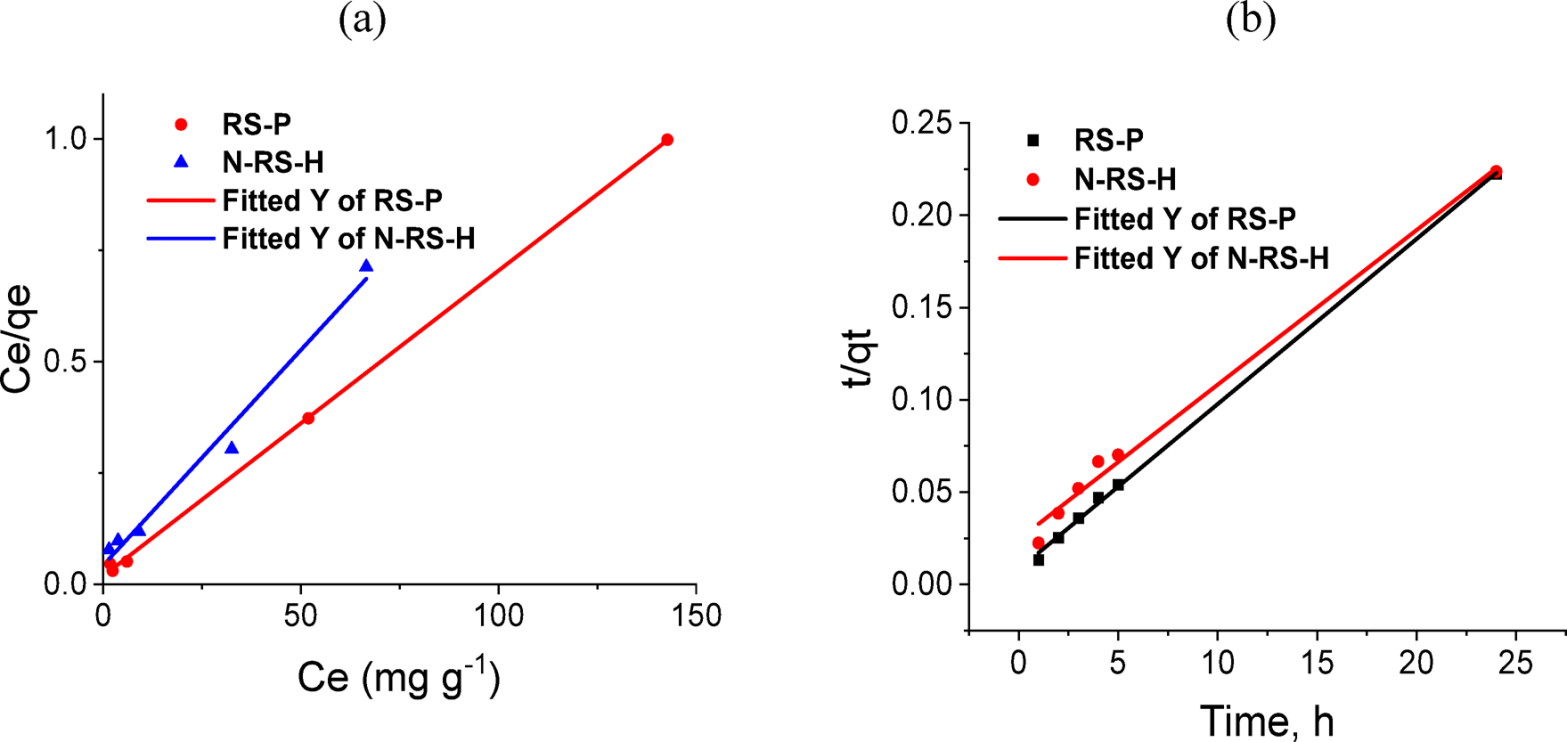
(**a**) Linear fitting plots of Langmuir isotherm and (**b**) Linear fitting plots of pseudo-second-order of optimum samples.

In contrast, the pseudo-second-order model provided an excellent fit for both adsorbents, with R^2^ values of 0.992 for N-RS-H and 0.999 for RS-P. The calculated equilibrium capacities (q_eq_) closely matched experimental values, confirming that chemisorption mediated by electron sharing or exchange between MB molecules and functional groups on the biochar surface is the primary adsorption mechanism. Notably, RS-P exhibited a higher rate constant (K₂ = 0.010 mg g⁻^1^ h⁻^1^) compared to N-RS-H (0.003 mg g⁻^1^ h⁻^1^), reflecting faster attainment of equilibrium. This difference likely arises from the dense acidic surface functionalities of RS-P, which provide abundant electrostatically active sites for immediate MB binding. In contrast, N-RS-H relies more on gradual intraparticle diffusion through its microporous and nitrogen-functionalized structure to achieve full adsorption. The intraparticle diffusion model provided further insight into the adsorption mechanism. Both biochar exhibited multi-stage adsorption processes, indicative of surface adsorption followed by gradual intraparticle diffusion into internal pores. N-RS-H displayed a higher intraparticle diffusion rate constant (Kᵢd = 15.98 mg g⁻^1^ h⁻½), highlighting rapid pore diffusion, whereas RS-P showed a larger intercept (C = 69.2 vs. 30.1 for N-RS-H), suggesting that external surface adsorption and boundary layer effects contribute more significantly to its kinetics. This observation aligns with the structural differences between the two biochar: N-RS-H possesses highly developed microporosity enhanced by nitrogen doping, while RS-P is dominated by surface-exposed acidic groups. These findings together show that the pseudo-second-order model successfully represents the adsorption kinetics of MB on both biochar. RS-P achieves fast first-stage adsorption because its surface contains many acidic sites but N-RS-H reaches peak capacities through the combined effects of surface diffusion and intraparticle diffusion mechanisms. Biochar design for dye adsorption requires understanding how surface chemistry interacts with pore structure because these two factors work together to determine performance. Figure 5 shows the linear fitting plots of the Langmuir adsorption isotherm and the pseudo-second-order kinetic model for the optimum biochar samples. The practical applicability of the prepared biochar adsorbents is justified by their high adsorption capacity, low cost, ease of regeneration, and environmental friendliness, making them a competitive alternative to conventional methods such as coagulation–flocculation, advanced oxidation, and membrane filtration.

### Elemental analysis

The elemental CHNO composition and derived atomic ratios provide important insights into the structural evolution and surface chemistry of the rice straw biochar and their correlation with adsorption capacity (Table 8). Pristine RS-biochar exhibited the highest carbon content (67.02%), indicative of a carbon-rich and aromatic structure, which aligns with its moderate adsorption capacity (Qm = 47.4 mg g⁻1) and specific surface area (S_MB_ = 150.6 × 10⁻3 km2 kg⁻1). In contrast, chemically modified samples, such as N-RS-H (60.34% C, 34.54% O) and RS-P (61.19% C, 35.98% O), showed reduced carbon but substantially higher oxygen contents, reflecting the incorporation of oxygenated functionalities during activation. These modifications coincided with a remarkable enhancement in adsorption capacity (Q_m_ = 136.9 ± 6.9 and 145.7 ± 7.3 mg/g, respectively) and surface area (S_MB_ = 434.9 and 462.8 × 10⁻3 km2 kg⁻1).

**Table 8:** CHNO for RS, N-RS-H, N-RS-M and RS-P biochars.

	C,%	H,%	N,%	O,%	H/C	O/C	C/N	(O + N)/C
RS-biochar	67.02	1.79	1.362	29.828	0.318	0.334	57.401	0.352
N-RS-H biochar	60.335	1.56	3.564	34.541	0.308	0.430	19.748	0.480
N-RS-M biochar	65.002	1.894	2.465	30.639	0.347	0.354	30.761	0.386
RS-P biochar	61.19	2.105	0.730	35.975	0.410	0.441	97.781	0.452

The H/C ratio, which indicates aromaticity and structural condensation, was lowest for RS (0.318) and N-RS-H (0.308), suggesting a higher degree of aromaticity and thermal stability. Despite this high aromaticity, N-RS-H exhibited a substantial increase in adsorption capacity, which can be attributed to its higher O/C ratio (0.430), indicating the enrichment of polar oxygenated groups that promote electrostatic interactions and hydrogen bonding with methylene blue. Conversely, RS-P displayed the highest H/C ratio (0.410) and O/C ratio (0.441), suggesting a less condensed but more polar structure. This high polarity is consistent with its superior surface area and adsorption capacity, confirming that phosphoric acid activation promotes both porosity development and oxygen functionalization ^69^.

Nitrogen incorporation was particularly evident in the N-doped biochar, with N-RS-H and N-RS-M containing 3.56% and 2.47% nitrogen, respectively, resulting in lower C/N ratios (19.75 and 30.76) compared with pristine RS (57.40). This confirms successful nitrogen functionalization, which introduces basic surface sites and enhances adsorption affinity toward cationic dyes. Notably, N-RS-M, despite its improved nitrogen content, showed only a moderate adsorption capacity (Q_m_ = 42.7 ± 2.13 mg/g) and surface area (S_MB_ = 135.5 × 10⁻3 km2 kg⁻1), emphasizing that the modification route plays a decisive role in tuning both porosity and functionality. By contrast, RS-P, with the lowest nitrogen content (0.73%) and highest C/N ratio (97.78), relied mainly on oxygen functionalization, which nonetheless delivered the highest adsorption performance. The (O + N)/C ratio further supports these trends, with N-RS-H (0.480) and RS-P (0.452) exhibiting the greatest heteroatom substitution, directly correlating with their superior adsorption capacities and specific surface areas. In comparison, RS (0.352) and N-RS-M (0.386) retained relatively higher carbon fractions with moderate heteroatom incorporation, which limited their adsorption performance. Based on the CHNO results, N-RS-H and RS-P exhibit superior adsorption performance due to their enhanced porosity and surface functionalization, whereas pristine RS remains carbon-rich but less reactive, and N-RS-M serves as an intermediate, reflecting the critical role of modification pathway and chemistry–porosity balance.

### FTIR analysis

**Fig. 6:**
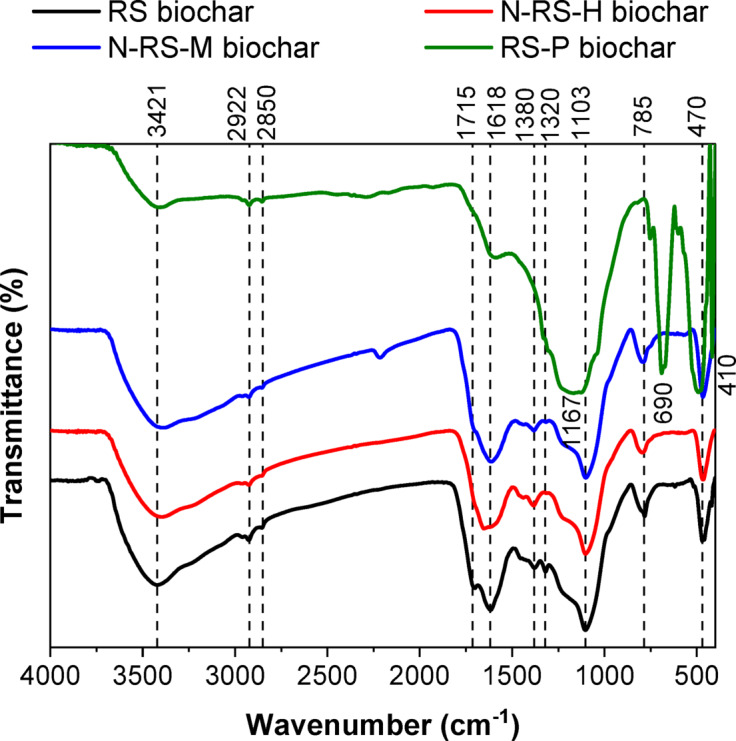
FTIR of RS, N-RS-H, N-RS-M and RS-P biochar.

The Fourier transform infrared (FTIR) spectra of rice straw–derived biochar (Fig. 6) revealed distinct vibrational bands corresponding to functional groups on the biochar surfaces, reflecting the influence of feedstock composition and chemical modifications. A broad band centered near 3420 cm⁻^1^ was attributed to –OH and N–H stretching vibrations, indicative of surface hydroxyl groups and incorporated nitrogen functionalities. Peaks in the 2922–2850 cm⁻^1^ region corresponded to aliphatic C–H stretching, representing residual cellulose/hemicellulose fragments and partially carbonized moieties. Distinct absorptions at ~ 1715 and 1650 cm⁻^1^ were assigned to C=O stretching in carboxyl, carbonyl, and amide groups, which can act as active adsorption sites for cationic molecules such as methylene blue (MB). Bands observed at 1380–1320 cm⁻^1^ were attributed to C–N stretching or O–H bending, further confirming nitrogen incorporation in N-doped samples. Strong absorption around 1110 cm⁻^1^ corresponded to C–O stretching of alcohols, ethers, and acids, with RS-P exhibiting additional P–O–C and P=O vibrations at 1167 cm⁻^1^, directly evidencing successful phosphorus doping. Bands below 800 cm⁻^1^, attributed to aromatic C–H out-of-plane bending, confirmed the presence of stable aromatic domains in all biochar. Notably, Si–O–Si stretching appeared consistently between 1080–1100 cm⁻^1^, with weaker bending modes near 460 cm⁻^1^, reflecting the high silica content of rice straw ash. The persistence of these Si–O–Si features in both pristine and modified biochar indicates structural stability and suggests that hydroxylated silicate species may contribute to MB adsorption through hydrogen bonding or electrostatic interactions ^63^.

Comparing pristine and chemically modified biochar revealed clear modifications in surface chemistry. RS-biochar displayed relatively weak –OH and C=O signals, consistent with a carbonized, less functionalized structure. Hydrothermally N-doped biochar (N-RS-H) showed intensified –OH/N–H and C–N/C=O bands, supporting the incorporation of nitrogen functionalities that enhance basic sites, electron-donor capacity, and potential π–π interactions. The red shift of the –OH/N–H band from 3420 to 3393 cm⁻^1^ further corroborates nitrogen incorporation. Microwave N-doping (N-RS-M) produced moderate enhancement, consistent with less efficient functionalization under milder conditions^57^. RS-P displayed pronounced P–O–C and P=O absorptions, along with a broadened –OH band at 1167 cm⁻^1^, indicative of an oxygen-rich and polar surface resulting from chemical activation. The additional low-frequency bands at 490 and 416 cm⁻^1^ appear to match the phosphate and silicate bending vibrations which indicates the development of new inorganic surface functionalities. The combination of these surface modifications creates a more polar environment in RS-P while increasing its heteroatom content which enables hydrogen bonding and electrostatic interactions and complexation mechanisms with cationic dyes.

### SEM analysis

**Fig. 7:**
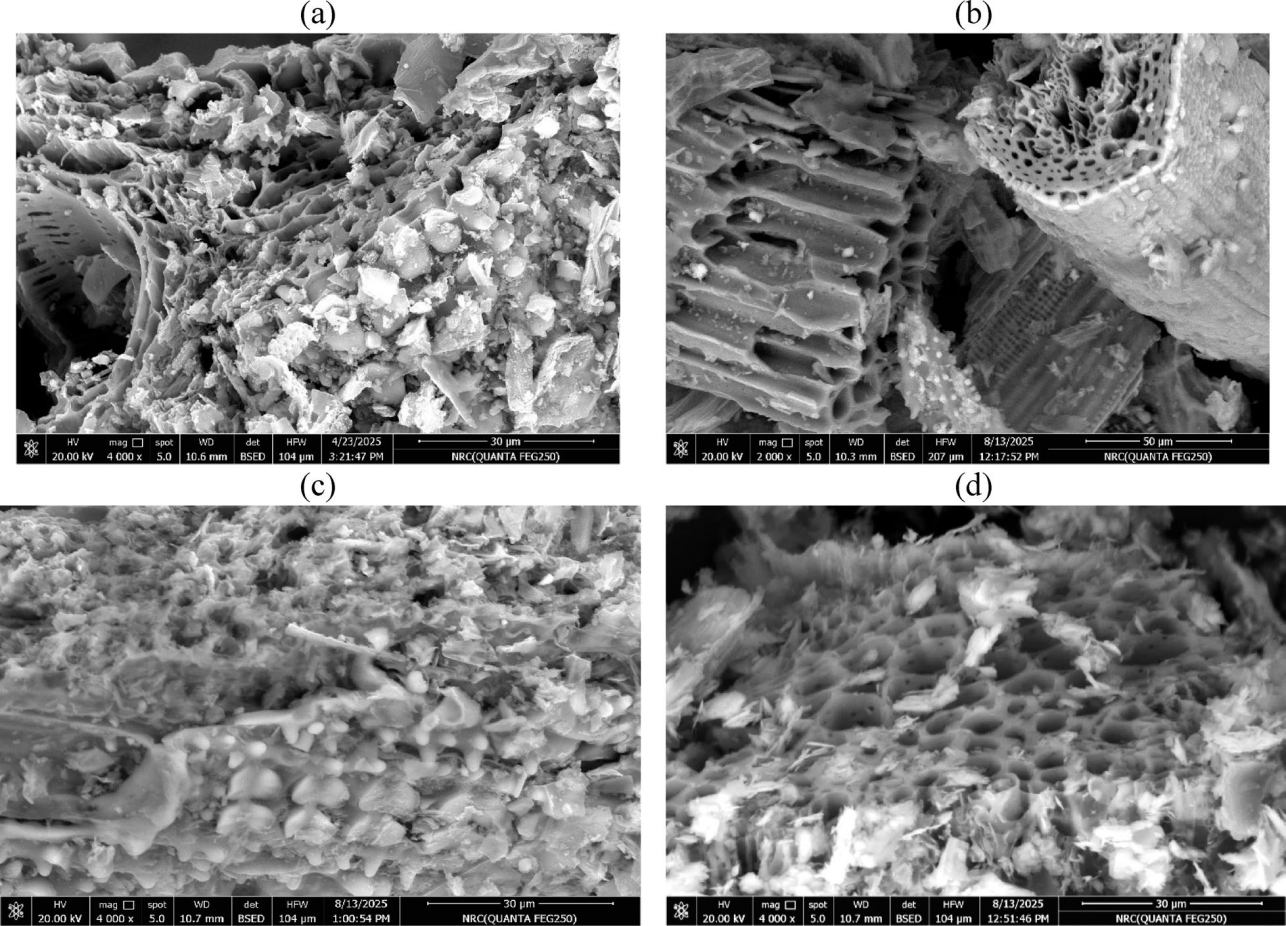
SEM images of (**a**) RS biochar, (**b**) N-RS-H biochar, (**c**) N-RS-M biochar and activated RS-P.

Figure 7 presents the SEM micrographs, which clearly demonstrate the distinct morphological transformations among the biochars, consistent with their specific surface area and MB adsorption capacities (Table 3). The pristine RS-biochar exhibits a compact, irregular surface with limited porosity, accounting for its relatively low specific surface area (150.6 × 10⁻^3^ km^2^ kg⁻^1^) and modest methylene blue (MB) adsorption capacity (Qm = 47.4 ± 2.4 mg g⁻^1^). In contrast, the N-RS-H biochar displays a rough and fragmented surface with abundant micro- and mesopores, resulting from nitrogen incorporation through the hydrothermal process. This enhanced textural development corresponds to its significantly higher surface area (434.9 × 10⁻^3^ km^2^ kg⁻^1^) and adsorption capacity (Qm = 136.9 ± 6.9 mg g⁻^1^), confirming that nitrogen doping and oxidative treatment introduced additional active sites that favor electrostatic and π–π interactions. The N-RS-M biochar, prepared under milder modification conditions, exhibits a partially developed porous framework with limited surface fragmentation. Its moderate surface area (135.5 × 10⁻^3^ km^2^ kg⁻^1^) and MB adsorption (Qm = 42.6 ± 2.1 mg g⁻^1^) reflect this intermediate degree of porosity and functionalization. Meanwhile, the RS-P biochar presents a highly developed sponge-like architecture with interconnected pore channels, characteristic of phosphoric acid activation involving dehydration and crosslinking reactions. Additionally, fine silicate particles are clearly visible on the surface, derived from the inherent silica content of rice straw. These silicate-rich domains not only enhance the mechanical stability of the matrix but may also contribute to adsorption via surface hydroxyl and silanol groups. The combination of phosphorus and silicon functionalities results in the highest specific surface area (462.8 × 10⁻^3^ km^2^ kg⁻^1^) and high Qm value (145.8 ± 7.3 mg g⁻^1^) confirm that phosphorus and oxygen functionalization effectively enhance both porosity and surface polarity, leading to improved dye adsorption performance.

## Conclusion

**Fig. 8:**
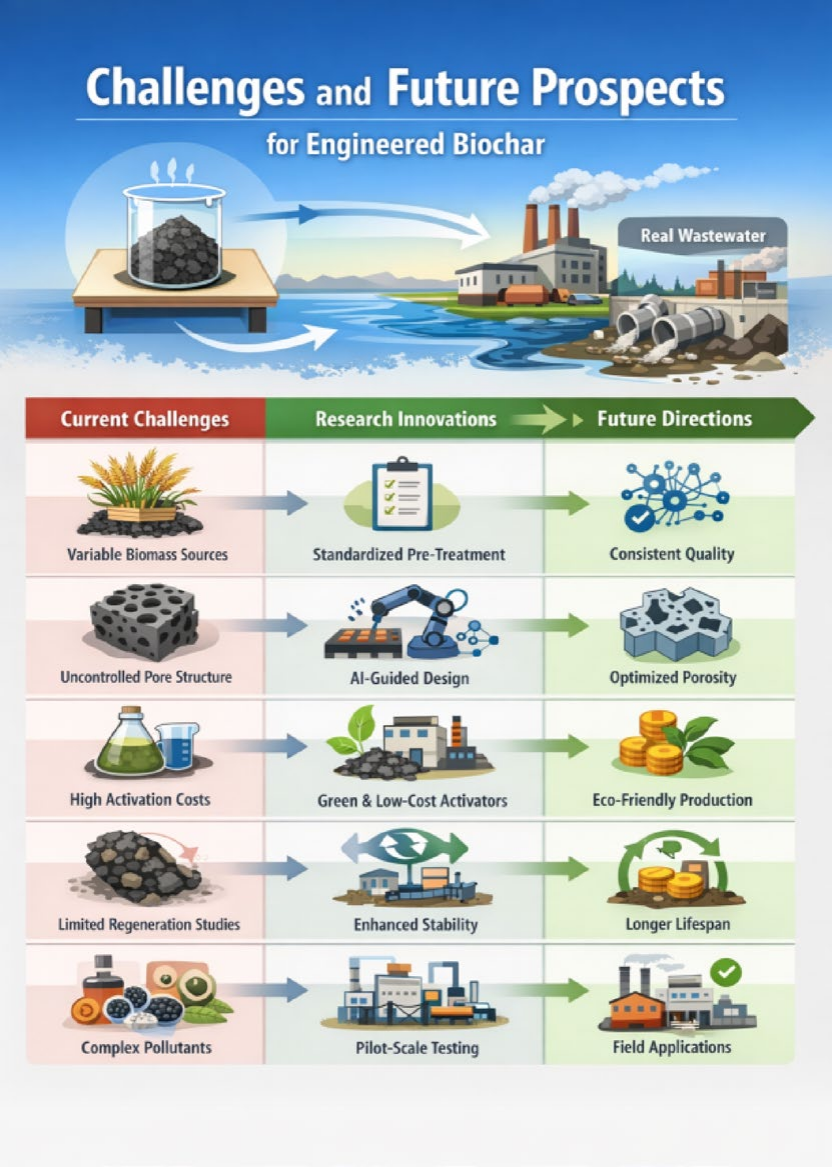
Challenges and future prospects (https://chatgpt.com/s/m_69ce31f3b7008191bda4366076b9d6e2).

This study shows that agricultural-residue biochar derived from four agricultural wastes (RS, DP, SCB and GR), with further enhancement via nitrogen doping and phosphoric-acid activation, provide high adsorption performance while promoting sustainability by valorizing low-cost wastes, minimizing environmental hazards, and enabling eco-friendly wastewater treatment. Among the pristine samples, RS-biochar showed high aromaticity but limited surface functionality, resulting in moderate adsorption performance. Hydrothermally N-doped RS (N-RS-H) significantly enhanced microporosity, nitrogen content, and basic surface sites, achieving a high adsorption capacity (Qm = 136.9 mg g⁻^1^) and large specific surface area (434.9 × 10^3^ m^2^ kg⁻^1^). Phosphoric-acid-activated RS (RS-P) developed a sponge-like porous structure enriched with oxygenated and silicate–phosphate groups, showing the highest surface area (462.8 × 10^3^ m^2^ kg⁻^1^) and maximum MB uptake (Qm = 145.8 mg g⁻^1^). Mechanistic analyses indicate that RS-P benefits mainly from enhanced porosity and acidic oxygen functionalities, whereas N-RS-H excels through nitrogen functionalities and surface basicity, both promoting efficient dye removal via hydrogen bonding, electrostatic interactions, and π–π stacking. All these findings, in comparison with literature, highlight two complementary and sustainable strategies (heteroatom functionalization and low cost chemical activator) for producing high-performance biochar, emphasizing the critical role of both surface chemistry and porosity in optimizing wastewater remediation. Future work would also benefit from graphical frameworks that map precursor selection, modification strategy, structure evolution, and adsorption mechanisms, providing visual guidance for rational biochar design (Fig. 8).

## Data Availability

All data are included in article.
